# Effectiveness and Safety of Treatment with Neodymium:YAG Laser 1064 nm in Patients with Classic and Epidemic Kaposi Sarcoma

**DOI:** 10.3390/bioengineering9030106

**Published:** 2022-03-05

**Authors:** Martina Silvestri, Alessandra Latini, Ilaria Lesnoni La Parola, Claudia Messina, Steven Paul Nisticò, Norma Cameli

**Affiliations:** 1 Unit of Dermatology, Department of Health Sciences, Magna Graecia University, 88100 Catanzaro, Italy; nistico@unicz.it; 2 Department of Dermatology, San Gallicano Dermatological Institute-IRCCS, 00144 Rome, Italy; alessandra.latini@ifo.it (A.L.); ilaria.lesnonilaparola@ifo.it (I.L.L.P.); claudia.messina@ifo.it (C.M.); norma.cameli@ifo.it (N.C.)

**Keywords:** YAG, classic Kaposi’s sarcoma, HIV, laser treatment

## Abstract

Kaposi’s sarcoma (KS) is a vascular neoplasm Herpes Virus 8 (HHV8), which can affect the skin, mucous membranes and viscera. There is currently no standard treatment for KS; this study evaluated the efficacy and safety of Neodymium:YAG (Nd:YAG) laser 1064 nm treatment in patients with classic and HIV-associated KS. 15 patients with classic KS (group A) and 15 with epidemic KS (group B), with exclusively cutaneous localization, were treated with Nd:YAG laser 1064 nm. Four treatment sessions were performed at 4 weeks intervals. 24/30 (80%) of treated patients underwent clinical improvement. Better results have been obtained in HIV-positive patients, especially in terms of reduced lesion size and the flattening of elevated lesions. The 1064 nm Nd:YAG laser is effective and safe in the treatment of classic and epidemic KS, especially in patients with symptomatic, slow-progressing local disease, where other treatment options may be inappropriate.

## 1. Introduction 

Kaposi sarcoma (KS) is an enigmatic vascular neoplasm that originates from the cells of the lymphatic endothelium and can involve the skin, mucous membranes and viscera. Kaposi sarcoma herpesvirus/human herpesvirus 8 (KSHV/HHV-8) is a necessary, but not sufficient, etiological factor for the development of this neoplasm [1]. There are four recognized epidemiological-clinical forms of KS, including classic or Mediterranean KS of the elderly, African (endemic) KS in children and young adults from Central Africa, iatrogenic KS (mainly in transplant patients, but also in patients undergoing chemotherapy or other immunosuppressive therapies) and epidemic KS, associated with HIV that is an AIDS-defining tumor [2]. KS lesions initially present as macules or patches (macular-patch stage), and subsequently evolve into plaque (plaque stage/plaque stage) and nodules (nodular-tumoral stage/nodular-tumor stage). There is currently no standard treatment for KS, but it depends on the extent and location of the lesions, as well as the clinical type of the disease [3]. Local therapies include different approaches, depending on the characteristics of the sarcoma, such as clinical monitoring, use of compression stockings for the prevention of lymphedema, surgery, cryotherapy, radiotherapy, intralesional chemotherapy and laser therapy [4,5]. To date, various types of lasers have been used to destroy KS lesions, including carbon dioxide (CO_2_), argon, and pulsed dye lasers [6]. This study evaluated the efficacy and safety of the neodymium: yttrium—aluminum—garnet (Nd:YAG) laser in 30 patients with classic and HIV-associated KS. Nd:YAG laser therapy is a beneficial therapeutic option, particularly in patients whose lesions are not so extensive or debilitating as to warrant potentially toxic systemic therapy [6]. In advanced disease, the Nd:YAG laser could be a potential and promising adjuvant treatment to systemic therapy for the management of particularly annoying or disabling skin lesions.

## 2. Material and Methods 

Thirty patients diagnosed with classic and epidemic KS, with exclusively cutaneous localization of the disease and who had never undergone specific therapies for KS, were retrospectively recruited in this study. Patients who fell into the other categories (extra-cutaneous localization, iatrogenic form, endemic form) were excluded. Of the 30 patients included in the study, 15 (13 Male, 2 Female) had classic KS (group A), aged between 64 and 76 years and with IA, IB, II staging according to the classification system proposed by Brambilla et al. in 2003 [7]; group B included 15 male patients with epidemic form of KS, aged between 33 and 51 and with staging (AIDS Clinical Trials Group—ACTG) T0, I0, S0. All group B patients were on antiretroviral therapy (HAART), immunologically reconstituted, with a CD4 T cell count >200 cells/mm^3^, and undetectable viremia (see Table 1). All patients performed laboratory investigations (complete blood count, urinalysis and liver and kidney function tests), which revealed no relevant abnormalities. Furthermore, the execution of instrumental investigations (chest x-ray, complete abdomen ultrasound, colonoscopy and gastroscopy) excluded the systemic involvement of the disease. Most of the patients had multiple lesions with a diameter between 0.3 and 11.1 cm. A representative lesion was selected for each patient (minimum diameter of 0.5 cm), whose size (largest diameter) and type (macula-papule-nodule-plaque) were assessed before treatment (T0) and 12 weeks after the last treatment session (T4).

All patients gave signed informed consent before the procedure. A total of 174 lesions underwent 4 sessions of Nd:YAG laser treatment (Synchro FT^®^; DEKA-Mela, Calenzano, Italy) using a 2.5–5 mm spot (depending on the lesion size), with a fluence of 120–140 J/cm^2^ and a double pulse duration of 3 milliseconds each with a delay of 20 milliseconds. The treatment was tolerable and did not require the use of local anesthetics. Each patient was re-evaluated and treated with a new laser session after 4 weeks, for a total of 4 treatment sessions. Patients’ response to treatment was assessed clinically by evaluating reduction in representative lesion diameter, reduction in total number of lesions, and flattening of elevated lesions. Before treatment (T0) and at each follow-up visit, photographs were taken using a digital camera.

## 3. Results

Complete response to treatment (CR) was defined by total regression of the representative lesion at T5. A partial response (RP) to treatment required the achievement of at least two of the following criteria: 50% reduction in the diameter of the representative lesion, 50% reduction in the total number of lesions, flattening of representative elevated lesions (papules, nodules, plaques). The non-achievement and increase in the previously listed parameters were defined as stable disease (SD) and disease progression (DP), respectively. Of the 30 patients, 24 (80%) showed clinical improvement, of which 8 patients (26.7%) achieved CR and 16 patients (53.3%) achieved RP; of the remaining 6 patients (20%) with no clinical response, 5 had stable disease (16.7%) and 1 (3.3%) had disease progression (see Table 2). Specifically, 13.3% of group A showed a complete clinical response versus 40% of group B. Disease progression, with an increase in the total number of lesions at T5 compared to T0, occurred in only 1 out of 30 patients (3.3%) belonging to group A. The median reduction in lesion diameter at T4 was 66% in group A vs. 87% in group B (*p* = 0.04), while the median reduction in the number of lesions at T4 compared to T0 was 62.5% in group A and 71.43% in group B. The flattening of the elevated lesions occurred in 55.6% of group A and 87.5% of group B. Of the patients who at T0 presented plantar nodular lesions, at T5, 75% were able to walk and carry out daily activities independently. 

No relevant side effects were observed in any patients, with the exception of mild hypotrophic scarring (three patients) and post-inflammatory hyperpigmentation (three patients).

## 4. Discussion

**Figure 1:**
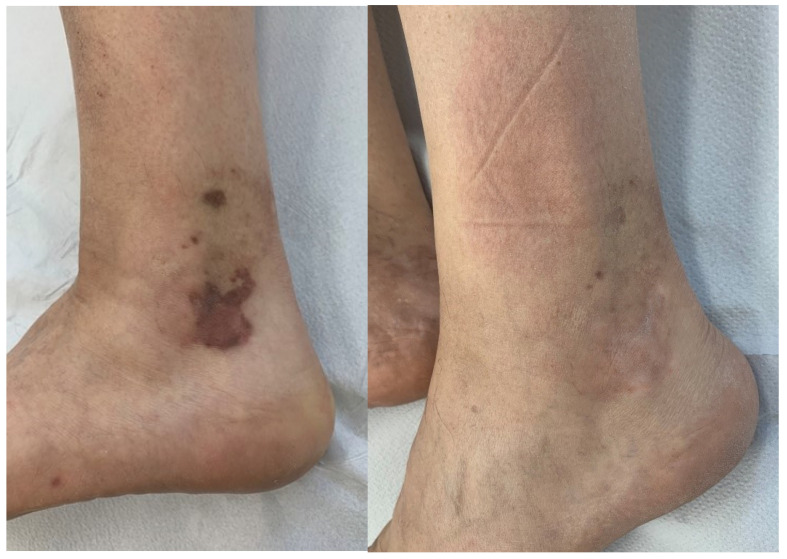
Patient with classic KS at T0 (**left**) and T5 (**right**).

**Figure 2:**
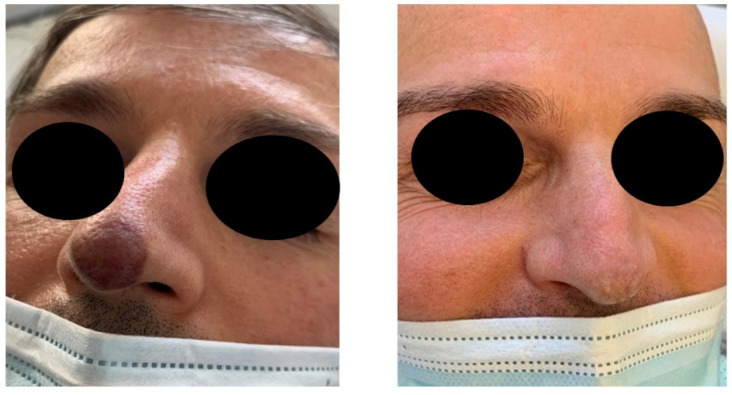
Patient with epidemic KS at T0 (**left**) and T5 (**right**).

In this retrospective study, the Nd:YAG laser 1064 nm, used for the treatment of classic and epidemic cutaneous KS, resulted in clinical improvement in 80% of treated patients (see Figure 1 and Figure 2).

Therapeutic options for the treatment of KS depend on the stage of the disease, the distribution of lesions, evolution, clinical type and immune status; however, no treatment provides a definitive cure, and the main goal of the various therapies remains the palliation of symptoms, in order to improve patients’ quality of life [8].

Asymptomatic lesions limited to the extremities, that do not cause functional alterations, are usually managed with compression stockings and periodic follow-up. Surgery, cryotherapy, radiotherapy, topical therapies with 9-cis-retinoic acid or imiquimod and intra-lesional chemotherapy, are the therapeutic choices commonly used in the case of localized and symptomatic lesions [9]. In HIV-positive patients, it is fundamental to improve the immune status. HAART alone can often lead to disease stabilization or remission in HIV-infected patients with slowly progressing cutaneous KS [10]. HAART, associated with concomitant systemic chemotherapy, should be reserved for patients with extensive and rapidly progressive cutaneous and visceral disease [11].

Several types of lasers have been used for the treatment of KS lesions, including CO_2_ laser, argon laser, Q-switched 755-nm Alexandrite laser, Pulsed-dye (PDL) laser, and Nd:YAG laser [12].

The Nd:YAG laser emits a light beam, with a wavelength of 1064 nm, targeting hemoglobin and deoxyhemoglobin, which allows it to penetrate deep vascular structures, causing minimal damage to the surrounding skin [13]. One of the limitations of laser therapy in the management of KS is the high relapse rate, resulting from insufficient skin penetration of the laser beam; this limit can be overcome by the use of high wavelengths, as in the case with the laser used in this study. Nasca et al. showed that the application of the laser beam, with an angle of inclination of 30°, 45° and 60° with respect to the skin, can increase the effectiveness of the treatment by focusing the laser beam on the vessels that are located in depth, maximizing the results and minimizing damage to the surrounding tissues [6]. Özdemir and Balevi treated seven patients with classical KS with the Nd:YAG laser, resulting in both clinical and histological improvement in all patients [14]. Laser therapy with Nd:YAG 1064 nm has also been shown to be effective in reducing the lymphedema associated with KS [15]. This beneficial effect may be due to the potential action of the laser beam on the cytokine pathways involved in the pathogenesis of KS; it is also conceivable that laser-induced hypoxia can cause an increase in Vascular Endothelial Growth Factor promoting the growth of lymphatic vessels with consequent lymphatic outflow [16]. This is the first study that evaluated the effectiveness of the Nd:YAG laser in the treatment of classical and epidemic KS, and the difference in response to treatment between the two groups. Patients with epidemic KS showed a better response to treatment than patients with classic KS, especially in terms of reduction in the mean diameter of the lesions and flattening of the elevated lesions. Numerous studies have attempted to identify the factors influencing the KS progression in HIV-infected patients, and HIV viral load control has been found to be essential for clinical improvement [16]; otherwise, there was no difference between the different types of antiretroviral drugs used (protease inhibitors versus integrase or non-nucleoside reverse transcriptase inhibitors) [17]. 

The limitations of the study were the small sample size and the short follow-up period. However, we believe these preliminary results are very promising and encourage the continued use of the therapeutic method evaluated in the study to confirm these results.

In this study, all group B patients were on HAART, immunologically recovered and with suppressed viral load. The Nd:YAG laser 1064 nm also showed a good safety profile, with no relevant side effects observed, with the exception of post-inflammatory hyperpigmentations and mild hypotrophic scars; moreover, no pain, crusting or continuous solutions were reported, because the laser beam leaves the epidermis intact, having little affinity for melanin.

## 5. Conclusions

The 1064 nm Nd:YAG laser is easy, effective and safe in the outpatient treatment of KS, in classic and epidemic cutaneous lesions. Its use appears to be very advantageous, especially in patients with symptomatic local disease, where other treatment options may not be applicable. In the treatment of KS, the 1064 nm Nd:YAG laser has the advantage of penetrating deeply into the tissues compared to other lasers. Currently, this is the first retrospective study comparing the efficacy of this laser treatment in patients with classic and epidemic KS, demonstrating that the 1064 nm Nd:YAG laser is effective in both forms of KS, providing better outcomes on cutaneous KS in HIV-positive patients. Furthermore, considering the safety profile and the low incidence of complications, this type of laser therapy can also be used as adjuvant therapy, in patients under systemic treatment with widespread disease.

## Figures and Tables

**Table 1:** Patient characteristics.

Patient	Age	Location	Diameter (cm)	HIV	Nodularity
1	68	Leg	1.7	No	No
2	70	Foot	0.8	No	Yes
3	69	Trunk	0.5	No	No
4	72	Leg	7.9	No	No
5	73	Foot	0.7	No	Yes
6	64	Thigh	5.3	No	No
7	76	Foot	0.5	No	Yes
8	71	Leg	0.6	No	No
9	65	Leg	6.4	No	No
10	67	Leg	11.1	No	No
11	75	Foot	0.5	No	Yes
12	73	Thigh	0.6	No	Yes
13	66	Trunk	10.5	No	No
14	70	Trunk	1.3	No	No
15	65	Leg	0.9	No	No
16	34	Trunk	0.5	Yes	No
17	45	Arms	5.8	Yes	No
18	50	Arms	0.9	Yes	Yes
19	44	Nose	0.6	Yes	No
20	40	Trunk	0.8	Yes	No
21	37	Arms	1.9	Yes	No
22	39	Trunk	0.9	Yes	No
23	47	Arms	2.6	Yes	No
24	46	Arms	1.9	Yes	Yes
25	50	Arms	0.8	Yes	Yes
26	38	Arms	2.2	Yes	No
27	49	Trunk	4	Yes	No
28	51	Arms	0.7	Yes	No
29	48	Arms	3.3	Yes	No
30	51	Trunk	4.1	Yes	No

**Table 2:** Response to treatment.

Clinical Response	Group A	Group B
	N (%)	N (%)
CR	2 (13.3)	6 (40.0)
PR	9 (60.0)	7 (46.7)
SD	3 (20.0)	2 (13.3)
DP	1 (6.7)	0

CR = complete response; PR = partial response; SD = stable disease; DP = disease progression.

## Data Availability

The data presented in this study are available on request from the corresponding author.
